# Using the behavior change wheel to design a novel home‐based exercise program for adults living with overweight and obesity: Comprehensive reporting of intervention development

**DOI:** 10.1002/osp4.774

**Published:** 2024-06-19

**Authors:** Sofie Power, David Broom, Michael Duncan, Stuart Biddle, Nikita Rowley

**Affiliations:** ^1^ Centre for Physical Activity Sport and Exercise Sciences Coventry University Coventry UK; ^2^ Centre for Health Research University of Southern Queensland Toowoomba Queensland Australia; ^3^ Faculty of Sport & Health Sciences University of Jyväskylä Jyväskylä Finland

**Keywords:** behavior change, home‐based exercise, obesity, overweight

## Abstract

**Introduction:**

Physical activity and exercise are movement behaviors that support the lifestyle management of overweight and obesity. However, home‐based exercise programs are commonly generic, and inconsistently undertake a holistic approach to program design.

**Methods:**

This work applied the Behavior Change Wheel, supplemented with previously conducted interviews, to the development of a home‐based exercise program, specifically for people living with overweight and obesity. This provided an understanding of the target behavior and identified a behavioral diagnosis. These findings were mapped onto the Capability, Opportunity, Motivation‐Behavior model and Theoretical Domains Framework, identifying changes needed and corresponding intervention functions.

**Results:**

Suitable Behavior Change Techniques were identified, alongside Capability Opportunity Motivation‐Behavior components needed to facilitate an increase in exercise behaviors, and five key intervention functions. This housed the delivery of 24 Behavior Change Techniques, including goal setting, feedback, monitoring and repetition.

**Conclusion:**

Applying the Behavior Change Wheel has enabled detailed development of a home‐based exercise program for adults living with overweight and obesity.

## Graphical abstract

Application of the behavior change wheel in the design and development of a home‐based exercise program for adults living with overweight and obesity. Twenty four suitable behavior change techniques were identified, alongside Capability, Opportunity and Motivation‐Behavior components and five intervention functions. This provided further direction for the future design of a home‐based exercise program specifically for adults living with overweight and obesity.

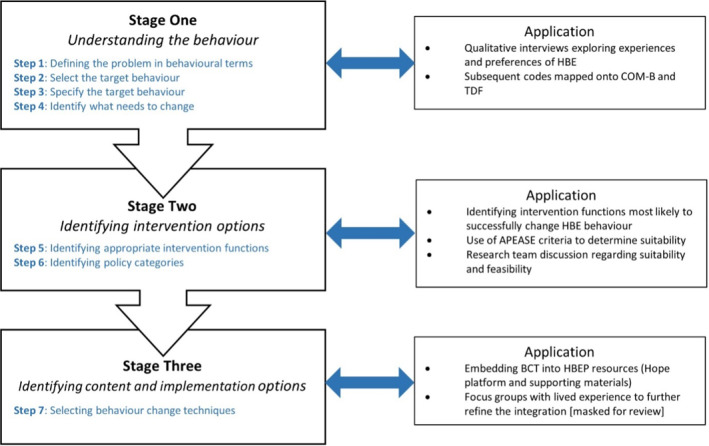

## 1 INTRODUCTION

Routinely, adults wishing to engage in physical activity and exercise behaviors do so through access to leisure facilities and community centers.^1^ However, specifically for adults living with overweight and obesity, exercising within the home may also be more comfortable, without the presence of exercise stigma or shame often present within a gym setting.^2^ Whilst the flexibility of home‐based exercise also suggests a suitable and popular alternative, there is limited evidence of the design of home‐based exercise programs tailored to the needs of adults living with overweight and obesity.^3^ The prescription of a generic exercise program lacks individualization for participants with additional health needs that may require a more needs‐sensitive program, where considerations are made for additional factors than just the immediate program content. This may include the exercise type, mode of delivery, or exercise intensity. This highlights the need for the design of a population‐specific home‐based exercise program specifically for adults living with overweight and obesity.

The design process of interventions encouraging behavior change should be theory informed,^4^ and consider the identification and incorporation of suitable Behavior Change Techniques (BCTs).^5^ Whilst the evidence for effectiveness of theory‐based interventions is mixed,^6^ as each selected theory may not contain all the necessary constructs to facilitate behavior change,^7^ there are many recommendations, including comprehensive reporting, contributing towards increased evidence.^8^ Despite the myriad of evidence of the benefits of physical activity and exercise for health, this is not reflected in adherence and engagement statistics where physical activity behavior has not improved since 2001.^9^ In addition, limited literature reports on the integration of a behavior change framework within home‐based exercise program design, specifically for adults living with overweight or obesity, further highlighting the need to further explore this aspect of program design and development.

**FIGURE 1:**
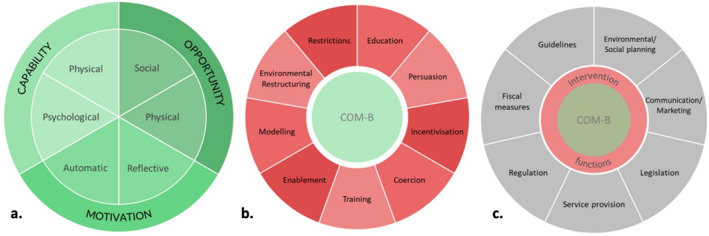
Segmented behavior change wheel. (A) The COM‐B components of the behavior change wheel; (B) intervention functions of the behavior change wheel; (C) policy categories of the behavior change wheel.

The Behavior Change Wheel (BCW) facilitates broad consideration of different behavioral options at different populations,^10^ preventing design commitment to a certain BCT without fully exploring different frameworks and techniques that may not have been considered otherwise. Figure 1 presents the BCW dissected further within Figures 1A–C. Specifically, regarding adults living with overweight and obesity and the current scarcity of needs‐sensitive home‐based exercise programs, a theoretical framework that prompts wide consideration of participants' needs would be deemed most appropriate to apply in this intervention design.

## 2 MATERIALS AND METHODS

### 2.1 Overview

**FIGURE 2:**
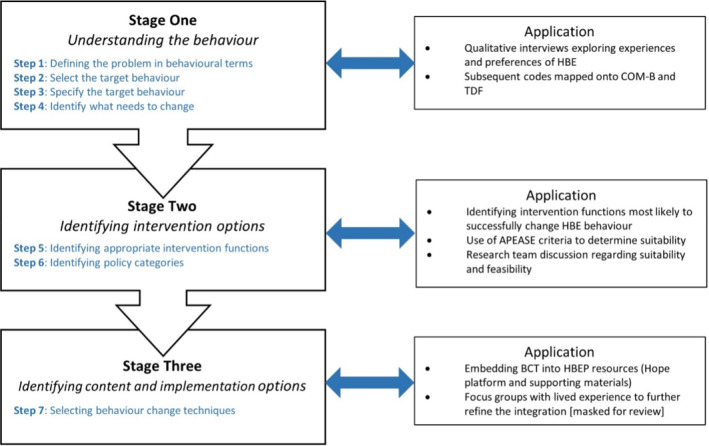
Overview and application of the method, with stages and steps from the BCW Blue text denotes steps as defined by Michie.^10^ APEASE criteria, Acceptability, Practicability, Effectiveness, Affordability, Spill‐over and Equity; BCW, Behavior Change Wheel; COM‐B, Capability, Opportunity, Motivation, Behavior; HBE, Home‐Based Exercise; HBEP, Home‐Based Exercise Program; TDF, Theoretical Domains Framework.

The BCW was applied to inform the development of a home‐based exercise program for adults living with overweight and obesity. It followed the application of three important stages, as previously defined,^10^ combined with evidence from current literature and qualitative data from previously conducted interviews with adults living with overweight and obesity.^11^ No ethics approval was required due to the nature of this secondary data work and for the supplementary interviews, ethics approval was granted by the Coventry University Ethics Review Committee (Reference: P123487). Figure 2 provides an overview and application of the method.

### 2.2 Stage one: Understanding the behavior

Stage one considered specific behavioral contexts of the problem, including the social and environmental context in which this behavior occurs as well as the individual, group, or population of focus. This included a review of relevant literature and exploring experiences and preferences of home‐based exercise for adults living with overweight or obesity through semi‐structured interviews, which have been previously reported within the literature.^11^ The identified themes and codes from the interviews were mapped onto the Capability, Opportunity, Motivation‐Behavior (COM‐B) model (displayed in Figure 1A), which informed the behavioral analysis and supported the consideration of BCTs to best encourage behavior change. This process allowed us to identify sources of behavior involved in exercise related choices specifically in adults living with overweight and obesity.

### 2.3 Stage two: Identifying intervention options

Following stage one, the BCW framework informed the chosen intervention functions, supported by literature, most likely to initiate behavior change within each COM‐B component. All intervention functions within the BCW and subsequently considered in this intervention development are displayed in Figure 1B.

The APEASE criteria (Affordability, Practicability, Effectiveness, Cost‐effectiveness, Acceptability, Side‐effects and Safety) were used to further determine the appropriateness of the chosen intervention functions. Supporting policy categories were then selected to maximize the potential of successful behavior change by enabling the chosen intervention functions to occur where links between intervention and policy categories were present.^12^ The policy categories in the BCW are displayed in Figure 1C.

### 2.4 Stage three: Intervention content

From the extensive list of 93 possible BCTs within the BCT Taxonomy v1,^13^ the authors selected techniques, justified by the previous stages detailed above, deemed to be most feasible and effective in eliciting the desired behavior change. The list was created by reviewing the evidence on effective techniques for increasing adults' physical activity and exercise,^14^ and mapping these BCTs to the intervention functions. The practical application and delivery of the intervention were discussed and thoroughly reviewed by the research team, identifying BCT implementation errors, and assessing their feasibility. Tracked changes were used to record each iteration of the subsequently developed program until a consensus was reached.

## 3 RESULTS

### 3.1 Stage one: Understanding the behavior

The identified target behavior was to increase exercise in adults aged 18–64 years living with overweight and/or obesity. The identified environment was the place of residence or the immediate vicinity such as the garden and/or driveaway, in accordance with the definition of home‐based exercise as previously defined.^15^ In line with global physical activity recommendations,^16^ achieving increased physical activity through a 12‐week home‐based exercise program was deemed an appropriate target behavior by the research team. This could be undertaken at any time of the day, where in program weeks 1–9 would be 3 times per week, weeks 10–12 would be 4 times per week. To further understand the behavior of home‐based exercise, experiences and preferences of home‐based exercise were previously explored through semi‐structured interviews.^11^ From this, the generated themes and codes that facilitated and impeded home‐based exercise were mapped against the COM‐B model, which can be seen in more detail in Supplementary Material one and the previously conducted semi‐structured interviews.^11^

*Psychological capability.* Both the interview data and the literature reported similar barriers to exercise. Participants reported that not knowing where to start was a key barrier, demonstrating similarity to existing and current research.^17^, ^18^

“*You know you can be over ambitious in what you're doing and actually end up hurting yourself and I don't want to do that. I want to stay as healthy as I can and* [my] *knowledge of exercise is not very great.”*

Possessing psychological capability, where in this work capability would be demonstrated through knowledge surrounding exercise, is important in making and sustaining behavioral change.^19^

Participants reported that a key facilitator of exercise was awareness of the psychological and physiological benefits of participation. Participants commented on the impact of this understanding on their exercise behavior, which is further reinforced within literature,^20^ where an awareness of the benefits of physical activity and exercise influences behavioral choice.

*Physical capability.* In the interview, a key barrier to exercise was the participant's physical exercise environment. Participants made reference to reduced physical space influencing their choice of, and engagement with, home‐based exercise. Similar findings were found within the literature, suggesting that individuals lacked sufficient space to be physically active, subsequently impeding exercise engagement.^21^ This may also link to the participant's psychological capability through reduced knowledge of how to exercise within their home environment.

*Physical opportunity.* Participants also reported that other responsibilities and commitments may take precedence over participation in exercise. Reduced time was a large barrier which would impact on engagement but conversely highlights home‐based exercise as a potential solution. One participant said:

“*Some of my other colleagues would go to the gym and I couldn’t be bothered to do it so I that's why I did it at home. Time wise it saves a lot time.*”

Further reinforced within the literature, the reduction in perceived, or actual, physical opportunity has been determined as a barrier to engagement in exercise.^22^ With consistent recognition that ‘lack of time’ could be an actual or perceived barrier by participants.

Another barrier to partaking in a home‐based exercise program was the physical environment in which the participants would conduct the exercise; not having sufficient space or the necessary equipment to conduct specific exercises were both barriers to participation. This recognition is also present within additional literature, similarly concluding that limited space and/or appropriate equipment does negatively impact exercise engagement.^23^

*Social opportunity.* Social support within exercise and physical activity has been seen as a facilitator of physical activity.^24^ Interview participants reported that social support from friends and family was a facilitator for engaging in a home‐based exercise. One participant demonstrated this through a relationship with their children and for some, this home environment offers participants social opportunity and subsequent increased engagement in home‐based exercise.^25^ However, for those that are isolated and use community‐based exercise for social opportunity, this may be a barrier to increasing exercise behavior.

Participants also reported the importance of a sense of community through undertaking group exercise, not only focusing on the exercise itself but also the opportunity to be part of an online/virtual community with others.

“If there's no social element to it and I can't talk to somebody, then I find that very difficult.”

The relationship between engaging in exercise and COM‐B categories, especially social support, is also recognized within the literature.^26^ Highlighting that a lack of social opportunity can be a barrier to exercise engagement.

*Reflective motivation.* Participants spoke of components in their lives that motivated them to undertake and continue exercise engagement. These motivators varied, but commonly included family members, fitness instructors and biofeedback from wearable technologies.

“*Having a watch that buzzes every half an hour and says move is no bad thing*.”

Similarly found within the literature, the result of biofeedback technologies encourages participants to lead a more active lifestyle, increasing awareness of their current lifestyle with a view for alteration.^27^

*Automatic motivation*. Enjoyment of exercise was identified as a key influence on exercise participation. One participant spoke of their enjoyment of exercise as the primary motivator for engagement. Receiving enjoyment through exercise contributed toward a positive feedback loop of continued exercise engagement, gradually making exercise habitual. This habitual, automatic motivation within the context of exercise has also been reported in the literature.^28^

These barrier and facilitator codes above have been mapped onto the COM‐B and further explored using the Theoretical Domains Framework (TDF), which can be seen in Table 1. This process facilitates comprehensive consideration within the selection and application of evidence‐based practice. The behavioral diagnostic process concluded that the most suitable components and domains were psychological and physical capability, social and physical opportunity, and automatic and reflective motivation.

**TABLE 1:** Further exploring the COM‐B and qualitative codes through TDF and identifying the needs to facilitate behavior change.

COM‐B component	Barrier (B) or facilitator (F) code(s)	Theoretical domains frameworks	Is there a need for change? If so, why?	What needs to happen for this change to occur?
Psychological capability	*Don't know where to start (B)* *More knowledge and guidance from a trusted source (F)*	Knowledge	**Change is needed** Participants may not have knowledge of specific exercises and correct technique.	Gain knowledge of program specific exercises and correct technique.
		Cognitive and interpersonal skills		
		Memory, attention and decision processes		Knowledge of the potential benefits of taking part in the HBEP.
		Behavioral regulation		
Physical capability	*Integration of technology (F)*	Physical skills	**Change is not needed**	
Physical opportunity	*Life getting in the way (B)* *The physical environment (B)* *Flexibility in accessibility (F)* *Lockdown restrictions as a result of COVID‐19 (F)*	Environmental context and resources	**Change is needed** Participant's physical environment may not facilitate HBEP.	Creation of opportunities for participants to be active at home.
Social opportunity	*Social support and those around them (F)* *Community, interactivity and relatedness (F)* *Virtual interactivity, social support and a team environment (F)*	Social influences	**Change is needed** Participants may lack a support network.	Create opportunities for participants to network with others on the HBEP for social support.
Reflective motivation	*Source of accountability and motivation (F)* *Progress monitoring and biofeedback* (F)	Social/professional role and identity	**Change may be needed** Some participants may not know the benefits of being physically active. Some participants may already know but do not, or are unable to, act upon their knowledge.	Believe that increasing physical activity is beneficial for their health.
		Beliefs about capabilities		
		Optimism		
		Intentions		Believe that they have the capability to undertake home‐based exercise.
		Goals		
		Beliefs about consequences		
Automatic motivation	*Enjoyment of the exercise* (F) *Variety is key* (F) *Uncontrollable barriers* (F)	Reinforcement Emotion	**Change is needed** Habit formation may not be established, preventing prolonged engagement in exercise.	Make engagement in the HBEP habitual.

Abbreviations: COM‐B, Capability, Opportunity, Motivation, Behavior; TDF, Theoretical Domains Framework.

### 3.2 Stage two: Identification of intervention options

The process of deeming intervention functions as suitable or non‐suitable was through using the APEASE criteria. Intervention functions deemed suitable were Education, Training, Modeling, Enablement and Environmental restructuring and therefore deeming Persuasion, Incentivisation, Coercion and Restriction as unsuitable and subsequently excluded. Further details on this process can be found in Supplementary Material two.

The selected intervention functions were also matched with the COM‐B and TDF most likely to be effective in changing exercise behavior. This can be found in Supplementary Material three.

Subsequently, the APEASE criteria was also used to explore suitable policy categories, alongside the intervention function and COM‐B components, as well as how this could be applied to a Home‐Based Exercise Program (HBEP). The outcome and application of this process are displayed in Table 2.

**TABLE 2:** Use of the APEASE criteria to identify suitable policy categories with complementary intervention functions and COM‐B components.

Intervention function	COM‐B component	Policy categories	Does the category meet the APEASE criteria? If so, how is it applied?
Education	Physical and psychological capability	Communication/marketing	Yes, this can encourage exercise. Printed and online materials can be incorporated.
	Reflective motivation	Guidelines	Yes, there will be a standardized HBEP for participants whilst also providing evidence to support the use of the program.
	Psychological capability, physical opportunity	Regulation	Yes, long‐term establishment of home‐based exercise principles of behavior and practice.
Training	Physical capability	Communication/marketing	Yes, current national guideline materials provide rationale for the HBEP.
		Guidelines	Yes, standardized HBEP will include guidelines that recommend practice.
	Physical opportunity	Environmental planning	Yes, individuals will create a space to exercise safely.
		Service provision	Yes, provide individuals with an exercise program and offer equipment and supervision.
	Social opportunity	Communication/marketing	Yes, provide opportunity to create a social network with other participants.
Modeling	Physical capability	Communication/marketing	Yes, communicate and regulate the exercise conducted and correct technique.
		Guidelines	Yes, detailed exercise instructions with imagery.
	Physical opportunity	Communication/marketing	Yes, communicate and regulate the exercise by modeling with correct technique.
Enablement	Psychological and physical capability	Communication/marketing	Yes, long‐term collective action to overcome barriers to adherence and participation.
	Social opportunity	Service provision	Yes, provide a platform for individuals to connect with others.
	Physical opportunity	Environmental planning	Yes, alter space to ensure exercise safety with addition of equipment if required.
	Automatic motivation	Communication	Yes, long‐term habit formation.
Environmental restructuring	Physical opportunity	Communication	Yes, encourage individuals to create a suitable environment for participation, with ease of access and without obstructions.
	Reflective motivation	Communication	Yes, encourage habit formation to reduce participant preparation effort.

Abbreviations: APEASE, Acceptability; Practicability, Effectiveness; Affordability, Spill‐over effects; and Equity; COM‐B, Capability; Opportunity, Motivation; Behavior; HBEP, Home‐Based Exercise Program.

### 3.3 Stage three: Intervention content

Within the five chosen intervention functions (education, modeling, enablement, training and environmental restructuring), a total of 24 BCTs were deemed appropriate by the research team, derived from the intervention development process and the appropriateness to the home‐based exercise program. The mode of delivery for these BCTs is via the HBEP delivered through Hope for the Community, an online self‐management platform for health interventions, providing easy access to participants, in which feasibility for other non‐communicable diseases have been demonstrated.^29^ Altogether, the Health at Home with Hope home‐based exercise program incorporates 24 BCTs across five intervention functions. The selected BCTs with official definitions and generic application to an exercise intervention are shown in Supplementary Material four. The BCTs and intervention functions correspond to COM‐B components, theoretical determinants, policy categories and HBEP‐specific intervention strategies. Table 3 shows the complete application of the BCW in the design of a HBEP specifically for adults living with overweight and obesity.

**TABLE 3:** Complete application of the BCW in the design of a HBEP specifically for adults living with overweight and obesity.

Intervention functions	COM‐B component	Theoretical determinants	BCTs with *implementation schedule*	Policy categories	Specific intervention strategy
Education	Psychological capability	Belief about consequences	Framing/reframing (13.2) *Week 0*	Communication/Marketing/guidelines	Appropriately frame messages about physical inactivity, exploring pros and cons of changing current exercise behavior.
	Reflective motivation	Decision processes	Pros and cons (9.2) *Weeks 0,6,13*	Communication/marketing	
	Reflective motivation	Knowledge	Information about health consequences (5.1) *Week 0*	Communication/marketing	Explain and explore potential short‐ and long‐term health consequences of not changing current exercise behavior.
	Social opportunity	Social influences	Credible source (9.1)^a^ *All*	Communication/marketing	HBEP to be built and delivered by a team at a higher education institution with appropriate qualifications.
	Reflective motivation	Optimism	Identity associated with changed behavior (13.5) *Week 0*	Communication	Emphasize a new identity associated with increased exercise behavior such as, increased confidence.
	Reflective motivation	Beliefs and capabilities	Feedback on outcomes of the behavior (2.7) *Weeks 6,13*	Communication/Guidelines	Provide feedback on outcomes of increasing exercise behavior.
	Automatic motivation	Reinforcement	Prompts/cues (7.1) *Weeks 1–12*	Communication/regulation	Prompts/cues to encourage engagement in the educational elements of the HBEP.
	Reflective motivation	Goals	Action planning (1.4) *Week 0*	Regulation	Prompt participants to consider how and when they will engage in the HBEP
Training	Reflective motivation	Goals	Goal setting (behavior) (1.1) *Week 0*	Regulation	Setting relevant behavioral goals to increase physical activity.
	Reflective motivation	Goals	Goal setting outcome (1.3) *Week 0*	Regulation	Setting relevant health outcomes goals to be achieved through increasing physical activity.
	Psychological capability	Behavioral regulation	Behavioral contract (1.8) *Weeks 0,6,12*	Regulation	Develop a contract of behaviors to increase exercise.
	Psychological capability	Intentions	Commitment (1.9) *Weeks 0,6,13*	Regulation	Consent to participating in the full HBEP.
	Automatic motivation	Goals	Reward (outcome) (10.10) *Weeks 6,13*	Marketing	Highlight the outcome reward of improved health upon completion.
	Physical capability	Physical skills	Demonstration of the behavior (6.1)^a^ *Weeks 0–12*	Communication/guidelines/service provision	Demonstrating and providing instruction of exercises to encourage good form and correct technique.
	Physical opportunity	Physical skills	Instruction on how to perform the behavior (4.1) *Weeks 0–12*	Communication/Guidelines/service provision	
	Psychological capability	Beliefs about capabilities	Behavioral practice/rehearsal (8.1) *Weeks 0–3*	Guidelines	Encourage exercise practice to improve confidence, skill and habit formation, reinforcing regular exercise importance.
	Psychological capability	Reinforcement	Habit formation (8.3) *Weeks 0,4,7,10*	Regulation	
	Physical capability	Physical skills	Graded tasks (8.7) *Weeks 0,4,7,10*	Regulation	Begin with easier exercises and provide suitable progression throughout.
Modeling	Physical capability/opportunity	Identity	Demonstration of the behavior (6.1)^a^ *Weeks 0–12*	Communication/guidelines/service provision	Demonstrate the exercises so individuals know correct technique.
	Psychological capability	Social influences	Credible source (9.1)* *All*	Communication/marketing/service provision	HBEP to be built and delivered by a team at a higher education institution with appropriate qualifications.
Enablement	Social opportunity	Social influences	Social reward (10.4) *Weeks 1–12*	Communication/social planning	Opportunity to share experiences and congratulations on progress.
	Physical opportunity	Environmental context and resources	Restructuring the physical environment (12.1)^a^ *Weeks 0,6*	Environmental planning	Enabling safe participation through restructuring the home environment such as, moving objects.
	Psychological capability	Identity	Identification of self as a role model (13.1) *Weeks 6–13*	Communication	Encourage individuals to see themselves as role models and consider how this may inspire others to make similar behavioral changes.
	Psychological capability	Reinforcement	Verbal persuasion about capability (15.1) *Weeks 0,6*	Communication	Reinforce participants’ ability to conduct the exercise and engage in the HBEP.
Environment restructuring	Psychological capability/Physical opportunity	Environmental context and resources	Problem solving (1.2) *Weeks 0,6*	Environmental planning	Enabling safe participation through restructuring the home environment, removing or changing objects or structures that may impact HBEP participation.
	Physical opportunity	Environmental context and resources	Restructuring the physical environment (12.1)^a^ *Weeks 0,6*	Environmental planning	
	Physical capability	Environmental context and resources	Adding objects to the environment (12.5) *Week 0*	Environmental planning	Provide participants with equipment to use throughout the HBEP.

Abbreviations: BCTs, Behavior Change Techniques; COM‐B, Capability, Opportunity, Motivation, Behavior; HBEP, Home‐Based Exercise Program.

^a^ Represents repeated BCTs under different intervention functions.

## 4 DISCUSSION

This study details the development of a home‐based exercise program for adults living with overweight and obesity using the BCW. The BCTs identified throughout the development process will supplement the design and delivery of a 12‐week online home‐based exercise program incorporating 24 BCTs across five different intervention functions. To date, the approach documented in this paper has not been implemented in the context of home‐based exercise program for adults living with overweight and obesity. Understanding this topic is key in creating sustainable exercise programs tailored to this specific population group. Consequently, the rigorous process outlined in the present study provides a template for researchers to follow for future intervention development.

Implementing the BCW during program design has supported the research team to consider the full array of techniques contributing toward program adherence, engagement and long‐term behavior change.^10^ The development of this complex program requires further consideration to just prescribing exercises,^30^, ^31^ and the BCW prompted exploration of different techniques that could facilitate that. It provided an opportunity to deeply consider and explore less obvious design features of the home‐based exercise program, to a further extent than what would have been undertaken without the application of the BCW. The inclusion of 24 BCTs across an intervention may be deemed impractical; however, due to the complex nature of this intervention, all 24 BCTs have a role within the HBEP. The identified BCTs also reflect those deemed to be more effective in recent systematic reviews within physical activity and exercise program design and prescription.^32^, ^33^ Even if BCTs had been considered during the design process, without application of the BCW, the subsequently identified BCTs could be predictable and commonly used in existing programs, of which the repetition may not be effective nor engaging for the participant. Therefore, the application of the BCW helped to consider techniques innovative and original to HBEP design.

The wider consideration and inclusion of different BCTs will also build a more well‐rounded, considered program, focusing on program engagement as a whole, rather than solely the prescribed exercise. Not only does this benefit participants by producing a program that aims to improve lifestyle behaviors but also defines the program as more than just an ‘exercise program’. Having determined that for people with lived experience, making positive lifestyle changes is bigger than just altering energy balance, developing a program that considers wider influences can be more effective than a program that solely prescribes exercise. Whilst all participants will have different circumstances and lifestyle considerations that will impact their ability to undertake and engage in a HBEP, designing a program that aims to account for these through BCTs provides an opportunity for participants to access a program that they can tailor to best suit their individual needs.

To the authors’ knowledge, the implementation of the BCW in the design and development of an HBEP for adults living with overweight and obesity is novel within the peer reviewed literature in this research area. It allows for further consideration of BCTs in comparison to exercise programs designed without, with scope to positively impact program engagement and adherence.

The detailed reporting of how the BCW was used within the programmed design for this home‐based exercise program throughout this study is a strength. There is limited literature available that comprehensively details the specific rationale and method of identifying and integrating BCTs into exercise interventions that include behavior change elements. Literature typically only reports the inclusion of BCTs but does not detail how or why those techniques were chosen. Without this, intervention development lacks robustness and decreases the chance of success when the program is implemented. Therefore, the detail in this paper contributes toward ease of repeatability by other researchers or health professionals looking to apply a theoretical behavior change framework within exercise program design as well as increased program success. It also provides the necessary detail to inform stakeholders, regardless of their specific intervention content, of the application and integration process of a behavior change framework within program design, subsequently improving the application and reporting of this process within the field.

Whilst we have tried to integrate appropriate BCTs into the design of the program, in order to account for lifestyle variability between participants, we recognize that not all the techniques will be appropriate and/or effective for all participants. Part of this process may also include a continual open dialogue with stakeholders in the program design and development process. This will allow for an iterative refinement process of the program and associated materials as well as evaluation of program aspects to further improve the output. Despite this, it can be challenging to develop a program that will suit a constantly growing population group, and therefore we understand that whilst this program aims to be helpful for the majority, we do recognize that participants may find elements of the program unhelpful. Whilst this may not be a limitation in its truest sense, the recognition of tailoring to different needs by researchers still remains important.

### 4.1 Future directions

Future program development research should aim to further tailor and develop home‐based exercise programs for adults living with overweight and obesity to facilitate maximum program flexibility and increase program engagement and adherence. This would include interaction with people with lived experience, both with regard to the content of the developed program itself and also how program flexibility and engagement can be simultaneously achieved.

We also widely encourage future use of a behavior change framework in the design of exercise programs. Whilst we recognize that the BCW may not be suitable for all intervention development processes, a behavioral framework would facilitate a wider consideration and a targeting of factors that influence participant engagement and adherence and subsequently, program success.

In addition to using a behavior change framework in intervention design, thorough reporting of the process should also be encouraged. Dissemination of the design process would aid researchers, developers and practitioners to learn about the whole process rather than just the finished project. For example, this may be through research team links to national and regional physical activity networks, local authorities, charities, and organizations that focus on obesity. The need for comprehensive reporting and choice justification, when using behavior change strategies, within intervention designs aligns with the work of Biddle et al.^34^ They have highlighted the current lack of explicit detail and justifications for the theoretical underpinnings of interventions, with even fewer stating how the chosen theory links to the behavior change strategies.

This work details the application of the BCW in the design and development of a home‐based exercise program for adults living with overweight and obesity. It specifies the implementation of a theoretical behavior change framework and explains how this contributes to an informed and considered program design. The research team encourages others to do the same when designing and implementing exercise programs. Specifically, within overweight and obesity, where long‐term program adherence and engagement are inconsistent, considering a behavioral change framework within the intervention design phase is likely to increase the chances of program success.

## AUTHOR CONTRIBUTIONS

Sofie Power, David Broom, Michael Duncan and Nikita Rowley developed the idea for this research. Sofie Power and Nikita Rowley conducted the behavioral analysis and subsequently interpreted and applied the results. All authors were involved in writing, editing and approving the submitted and final manuscript.

## CONFLICT OF INTEREST STATEMENT

The authors declare that there is no conflict of interest that has influenced this research at any stage.

## Supporting information

Table S1

Table S2

Table S3

Table S4

## Data Availability

The current article is accompanied by the relevant data generated during and/or analyzed during the study, including the complete database of Behavior Change Wheel application. These files are available and accessible as Supplemental Material. Verbatim interview transcripts are not publicly available to maintain participant anonymity, but anonymized transcripts are available from the corresponding author upon reasonable request.

## References

[1] ukactive . Moving Communities: Active Leisure Trends 2019 Report; 2019:1‐24. Published online. www.datahubclub.com

[2] Thedinga HK , Zehl R , Thiel A . Weight stigma experiences and self‐exclusion from sport and exercise settings among people with obesity. BMC Publ Health. 2021;21(1):565. 10.1186/s12889-021-10565-7 PMC7983352 33752645

[3] Power S , Rowley N , Flynn D , Duncan M , Broom D . Home‐based exercise for adults with overweight or obesity: a rapid review. Obes Res Clin Pract. 2022;16(2):97‐105. 10.1016/j.orcp.2022.02.003 35183471 PMC9817080

[4] Skivington K , Matthews L , Simpson SA , et al. A new framework for developing and evaluating complex interventions: update of Medical Research Council guidance. BMJ. 2021;374:n2061. 10.1136/bmj.n2061 34593508 PMC8482308

[5] Harrison AL , Taylor NF , Shields N , Frawley HC . Attitudes, barriers and enablers to physical activity in pregnant women: a systematic review. J Physiother. 2018;64(1):24‐32. 10.1016/j.jphys.2017.11.012 29289592

[6] Davis R , Campbell R , Hildon Z , Hobbs L , Michie S . Theories of behavior and behavior change across the social and behavioural sciences: a scoping review. Health Psychol Rev. 2014;9(3):323‐344. 10.1080/17437199.2014.941722 25104107 PMC4566873

[7] Cane J , O’Connor D , Michie S . Validation of the theoretical domains framework for use in behavior change and implementation research. Implement Sci. 2012;7(37):37. 10.1186/1748-5908-7-37 22530986 PMC3483008

[8] Prestwich A , Webb TL , Conner M . Using theory to develop and test interventions to promote changes in health behavior: evidence, issues, and recommendations. Curr Opin Psychol. 2015;5:1‐5. 10.1016/j.copsyc.2015.02.011

[9] World Health Organization . Physical Activity ‐ Key Facts; 2020. Accessed 2 June 2021. https://www.who.int/news‐room/fact‐sheets/detail/physical‐activity

[10] Michie S , Atkins L , West R . The Behavior Change Wheel: A Guide to Designing Interventions. Silverback Publishing; 2014.

[11] Power S , Rowley N , Duncan M , Broom D . “I was having my midlife fat crisis”: exploring the experiences and preferences of home‐based exercise programs for adults living with overweight and obesity. Int J Environ Res Publ Health. 2022;19(19):12831. 10.3390/ijerph191912831 PMC9566702 36232130

[12] Michie S , van Stralen MM , West R . The behavior change wheel: a new method for characterising and designing behavior change interventions. Implement Sci. 2011;6(42):42. 10.1186/1748-5908-6-42 21513547 PMC3096582

[13] Michie S , Richardson M , Johnston M , et al. The behavior change technique Taxonomy (v1) of 93 hierarchically clustered techniques: building an international consensus for the reporting of behavior change interventions. Ann Behav Med. 2013;46(1):81‐95. 10.1007/S12160-013-9486-6 23512568

[14] Samdal GB , Eide GE , Barth T , Williams G , Meland E . Effective behavior change techniques for physical activity and healthy eating in overweight and obese adults; systematic review and meta‐regression analyses. Int J Behav Nutr Phys Activ. 2017;14(1):42. 10.1186/s12966-017-0494-y PMC5370453 28351367

[15] Denton F , Power S , Waddell A , et al. Is it really home‐based? A commentary on the necessity for accurate definitions across exercise and physical activity programs. Int J Environ Res Publ Health. 2021;18(17):9244. 10.3390/IJERPH18179244 PMC8431042 34501833

[16] Bull FC , Al‐Ansari SS , Biddle S , et al. World Health Organization 2020 guidelines on physical activity and sedentary behavior. Br J Sports Med. 2020;54(24):1451‐1462. 10.1136/bjsports-2020-102955 33239350 PMC7719906

[17] Kendrick J , Ritchie M , Andrews E . Exercise in individuals with CKD: a focus group study exploring patient attitudes, motivations, and barriers to exercise. Kidney Med. 2019;1(3):131‐138. 10.1016/J.XKME.2019.03.004 32705080 PMC7377257

[18] Jones K , Naisby J , Baker K , Tew GA . Exercise perceptions and experiences in adults with Crohn’s disease following a combined impact and resistance training program: a qualitative study. Crohns Colitis. 2023;5(2):1‐12. 10.1093/crocol/otad019 PMC10071982 37025161

[19] Flannery C , McHugh S , Anaba AE , et al. Enablers and barriers to physical activity in overweight and obese pregnant women: an analysis informed by the theoretical domains framework and COM‐B model. BMC Pregnancy Childbirth. 2018;18(1):178. 10.1186/S12884-018-1816-Z/TABLES/3 29783933 PMC5963099

[20] Leone LA , Ward DS . A mixed methods comparison of perceived benefits and barriers to exercise between obese and nonobese women. J Phys Activ Health. 2013;10(4):461‐469. 10.1123/jpah.10.4.461 PMC3904548 23714626

[21] Schneider C , Reimann S , Schmid J , et al. Qualitative analysis of facilitators and barriers to centre‐ and home‐based exercise training in breast cancer patients ‐ a Swiss tertiary centre experience. Swiss Med Wkly. 2022;152(19‐20):1‐15. 10.4414/smw.2022.w30155 35633635

[22] Cavallini FM , Callaghan ME , Premo CB , Scott JW , Dyck DJ . Lack of time is the consistent barrier to physical activity and exercise in 18 to 64 year‐old males and females from both South Carolina and Southern Ontario. J Phys Act Res. 2020;5(2):100‐106. 10.12691/jpar-5-2-6

[23] Farah BQ , do Prado WL , Malik N , et al. Barriers to physical activity during the COVID‐19 pandemic in adults: a cross‐sectional study. Sport Sci Health. 2021;17(2):441‐447. 10.1007/s11332-020-00724-5 33815618 PMC7998080

[24] Carter A , Alexander AC . “It’s a whole different atmosphere”: a qualitative examination of social support as a facilitator of exercise during the COVID‐19 pandemic. Health Promot Pract. 2021;22(5):622‐630. 10.1177/15248399211013005 33955244

[25] Bachmann C , Oesch P , Bachmann S . Recommendations for improving adherence to home‐based exercise: a systematic review. Phys Med. 2018;28(1):20‐31. 10.1055/S-0043-120527/ID/R2017-05-0265-0022/BIB

[26] Teo JL , Zheng Z , Bird SR . Identifying the factors affecting ‘patient engagement’ in exercise rehabilitation. BMC Sports Sci Med Rehabil. 2022;14(1):18. 10.1186/s13102-022-00407-3 35130940 PMC8819209

[27] Shin G , Feng Y , Jarrahi MH , Gafinowitz N . Beyond novelty effect: a mixed‐methods exploration into the motivation for long‐term activity tracker use. JAMIA Open. 2019;2(1):62‐72. 10.1093/jamiaopen/ooy048 31984346 PMC6952057

[28] Hagberg LA , Lindahl B , Nyberg L , Hellénius ML . Importance of enjoyment when promoting physical exercise. Scand J Med Sci Sports. 2009;19(5):740‐747. 10.1111/j.1600-0838.2008.00844.x 18694433

[29] Wright H , Martin F , Clyne W , et al. A digital self‐management program (help to overcome problems effectively) for people living with cancer: feasibility randomized controlled trial. J Med Internet Res. 2021;23(11). 10.2196/24264 PMC8726569 34738912

[30] Power S , Rowley N , Duncan M , Broom D . Co‐designing and refining a home‐based exercise programme for adults living with overweight and obesity: insight from people with lived experience. Obesities. 2023;3(2):132‐145. 10.3390/obesities3020011

[31] Bagnall AM , Radley D , Jones R , et al. Whole systems approaches to obesity and other complex public health challenges: a systematic review. BMC Publ Health. 2019;19(8):8. 10.1186/s12889-018-6274-z PMC6318991 30606173

[32] Carraça E , Encantado J , Battista F , et al. Effective behavior change techniques to promote physical activity in adults with overweight or obesity: a systematic review and meta‐analysis. Obes Rev. 2021;22(S4). 10.1111/obr.13258 PMC8365685 33949778

[33] Patterson K , Davey R , Keegan R , Kunstler B , Woodward A , Freene N . Behavior change techniques in cardiovascular disease smartphone apps to improve physical activity and sedentary behavior: systematic review and meta ‐ regression. Int J Environ Res Publ Health. 2022;19(81):1‐14. 10.1186/s12966-022-01319-8 PMC9261070 35799263

[34] Biddle SJH , Mutrie N , Gorely T , Faulkner G . Psychology of Physical Activity. 4th ed. Routledge; 2021. 10.4324/9781003127420

