# Supplementary material for: Using the behavior change wheel to design a novel home‐based exercise program for adults living with overweight and obesity: Comprehensive reporting of intervention development
Source: Obes Sci Pract. 2024 Jun 19;10(3):e774. doi: 10.1002/osp4.774 (PMC11187404; doi:10.1002/osp4.774)
Supplement: Supplementary file 1 — Table S1 [file OSP4-10-e774-s004.docx]

| **COM-B category** | **Relevant codes** | **Barrier or facilitator?** |
| --- | --- | --- |
| Psychological capability | *Don’t know where to start* | Barrier |
|  | *More knowledge and guidance* *from a trusted source* | Facilitator |
| Physical capability | *Integration of technology* | Facilitator |
| Social opportunity | *Social support and those around them* | Facilitator |
|  | *Community, interactivity and relatedness* | Facilitator |
|  | *Virtual interactivity, social support and a team environment* | Facilitator |
| Physical opportunity | *Life getting in the way* | Barrier |
|  | *Physical environment* | Barrier |
|  | *Flexibility in accessibility* | Facilitator |
|  | *Lockdown restrictions as a result of Covid-19* | Facilitator |
| Reflective motivation | *Source of accountability and motivation* | Facilitator |
|  | *Progress monitoring and biofeedback* | Facilitator |
| Automatic motivation | *Enjoyment of the exercise* | Facilitator |
|  | *Variety is key* | Facilitator |
|  | *Uncontrollable barriers* | Barrier |

Supplementary Material one

**Table 1**: Interactions between COM-B and interview codes, classified as barriers or facilitators.
