# Supplementary material for: Using the behavior change wheel to design a novel home‐based exercise program for adults living with overweight and obesity: Comprehensive reporting of intervention development
Source: Obes Sci Pract. 2024 Jun 19;10(3):e774. doi: 10.1002/osp4.774 (PMC11187404; doi:10.1002/osp4.774)
Supplement: Supplementary file 2 — Table S2 [file OSP4-10-e774-s002.docx]

Supplementary material two

**Table 2**: Use of the APEASE criteria to identify suitable intervention functions.

| **Potential intervention functions** | **Does the intervention meet the APEASE criteria?** | **Definitions of incorporated Intervention functions** |
| --- | --- | --- |
| Education | Yes, to be incorporated | Increasing knowledge or understanding |
| Persuasion | Not appropriate  *Inducing negative feelings to stimulate action was deemed inappropriate* |  |
| Incentivisation | Not affordable  *Limited project funding eliminates this intervention function* |  |
| Coercion | Not appropriate  *Implementing expectation of punishment or cost was deemed inappropriate* |  |
| Training | Yes, to be incorporated | Imparting skills |
| Restriction | Not relevant  *Using rules to restrict other competing behaviours was deemed irrelevant in this work* |  |
| Environmental restructuring | Yes, to be incorporated | Changing the physical or social context |
| Modelling | Yes, to be incorporated | Providing an example for people to aspire to or imitate |
| Enablement | Yes, to be incorporated | Increasing means/reducing barriers to increase capability or opportunity |
| Selected intervention functions | **Education, Training, Modelling, Enablement, Environmental restructuring** | |

APEASE: Acceptability, Practicability, Effectiveness, Affordability, Spill-over effects, and Equity

Italicised text denotes research team justification for not meeting the APEASE criteria.
