# Supplementary material for: Using the behavior change wheel to design a novel home‐based exercise program for adults living with overweight and obesity: Comprehensive reporting of intervention development
Source: Obes Sci Pract. 2024 Jun 19;10(3):e774. doi: 10.1002/osp4.774 (PMC11187404; doi:10.1002/osp4.774)
Supplement: Supplementary file 3 — Table S3 [file OSP4-10-e774-s003.docx]

Supplementary Material four

**Table 4**: Selected BCTs with official definition and generic application to an exercise intervention.

| **BCTs** | **BCT definition** | **General Exercise Intervention Strategy** |
| --- | --- | --- |
| Framing/  reframing (13.2) | *Suggest the deliberate adoption of a perspective or new perspective on behaviour in order to change cognitions or emotions about performing the behaviour.* | Appropriately frame messages about the associated risks of not changing behaviour (13.2) |
| Information about health consequences (5.1) | *Provide information about health consequences of performing the behaviour.* | Information about consequences to health if behaviour doesn’t change (5.1) |
| Credible source (9.1)* | *Present verbal or visual communication from a credible source in favour of or against the behaviour.* | HBEP delivered by credible sources – Coventry University, Exercise Scientists and Psychologists (9.1.) |
| Pros and cons (9.2) | *Advise the person to identify and compare reasons for wanting and not wanting to change the behaviour.* | Explore pros and cons of changing behaviour (9.2) |
| Identity associated with changed behaviour (13.5) | *Advise the person to construct a new self- identity as someone who ‘used to engage with the unwanted behaviour’* | Emphasis identity which can be associated with changed behaviour - increase confidence, etc. (13.5) |
| Feedback on outcomes of the behaviour (2.7) | *Monitor and provide feedback on the outcome of performance of the behaviour.* | Provide feedback on outcomes of changing behaviour (2.7) |
| Prompts/cues (7.1) | *Introduce or define environmental or social stimulus with the purpose of prompting or cueing the behaviour.* | Include prompts/cues to encourage individual to engage in the educational elements of the intervention (7.1) |
| Self-monitoring of behaviour (2.3) | *Establish a method for the person to monitor and record their behaviour(s) as part of a behaviour change strategy.* | Include options to self-monitor behaviour via fitness tracker (2.3) |
| Action planning (1.4) | *Prompt detailed planning of performance of the behaviour.* | Use of FITT principle throughout programme design and encourage participants to plan their HBEP engagement (1.4) |
| Goal setting (behaviour) (1.1) | *Set or agree on a goal defined in terms of the behaviour to be achieved.* | Setting relevant goals in terms of behaviours to increase PA (1.1) |
| Goal setting outcome (1.3) | *Set or agree on a goal defined in terms of a positive outcome* *of wanted behaviour.* | Goals setting in terms of health outcomes (1.3) |
| Behavioural contract (1.8) | *Create a written specification of the behaviour to be performed, agreed on by the person, and witnessed by another.* | Develop a contract of expected behaviours (1.8) |
| Commitment (1.9) | *Ask the person to affirm or reaffirm statements indicating commitment to change the behaviour.* | Commitment statement – consent to participating for full programme (1.9) |
| Reward (outcome) (10.10) | *Arrange for the delivery of a reward if and only if there has been effort and/or progress in achieving the behavioural outcome.* | Outcome reward in the form of weight loss, improved BP, etc. upon completed (10.10) |
| Demonstration of the behaviour (6.1)* | *Provide an observable sample of the performance of the behaviour, directly in person or indirectly.* | Demonstrating and providing instruction of behaviours/exercises to encourage good form and show individuals how to conduct each exercise correctly (6.1 & 4.1). |
| Instruction on how to perform the behaviour (4.1) | *Advise or agree on how to perform the behaviour.* | Demonstrating and providing instruction of behaviours/exercises to encourage good form and show individuals how to conduct each exercise correctly (6.1 & 4.1 |
| Behavioural practice/rehearsal (8.1) | *Prompt practice or rehearsal of the performance of the behaviour one or more times in a context or at a time when the performance may not be necessary, in order to increase habit and skill.* | Encourage individuals to practice the exercises to improve confidence (8.1) |
| Graded tasks (8.7) | *Set easy-to-perform tasks, making them increasingly difficult, but achievable, until behaviour is performed.* | Graded tasks – begin easy and provide suitable progression (8.7) |
| Habit formation (8.3) | *Prompt rehearsal and repetition of the behaviour in the same context repeatedly so that the context elicits the behaviour.* | Create a habit – reinforce the importance of daily exercise (8.3) |
| Demonstration of the behaviour (6.1)* | *Provide an observable sample of the performance of the behaviour, directly in person or indirectly.* | Demonstrate the exercises so individuals know how to perform them correctly (6.1) |
| Credible source (9.1)* | *Present verbal or visual communication from a credible source* *in favour of or against the behaviour.* | HBEP delivered by credible sources – Coventry University, Exercise Scientists and Psychologists(9.1.) |
| Social reward (10.4) | *Arrange verbal or non-verbal reward if and only if there has been* *effort and/or progress in performing the behaviour.* | Enable individuals to adhere to the programme. Social reward – networking with people who are also living with overweight and obesity – shared experiences (10.4) |
| Restructuring the physical environment (12.1)* | *Change, or advise to change the physical environment in order to facilitate performance of the wanted behaviour or create barriers to the unwanted behaviour.* | To enable individuals to participate, support the reconstruction of their environment to enable safe exercise (moving objects out of the way, etc.) (12.1) |
| Identification of self as a role model (13.1) | *Inform that one's own behaviour may be an example to others.* | Improve identity – as programme commences, encourage individuals to see themselves as role models if programme is going successful (increasing PA, improving health outcomes) (13.1) |
| Verbal persuasion about capability (15.1) | *Tell the person that they can successfully perform the wanted behaviour, arguing against self-doubts and asserting that they can and will succeed.* | Reinforce their ability to conduct the exercise (15.1) |
| Problem solving (1.2) | *Analyse, or prompt the person to analyse, factors influencing the behaviour and generate or select strategies that include overcoming barriers and/or increasing facilitators* | Problem solving relates to creating a space (environment restructuring) to exercise safely (1.2) |
| Restructuring the physical environment (12.1)* | *Change, or advise to change the physical* *environment in order to facilitate performance of the wanted behaviour or create barriers to the unwanted behaviour.* | Reconstructing the physical environment so that individuals have space to exercise (12.1) |
| Adding objects to the environment (12.5) | *Add objects to the environment in order to facilitate performance of the behaviour.* | Provide individuals with equipment for specific exercises (12.5) |

BCTs: Behaviour change Techniques; HBEP: Home-Based Exercise Programme; FITT: Frequency, Intensity, Type, Time

*Represents repeated BCTs under different intervention functions.

BCT Definitions from Michie^21^
