# Supplementary material for: Using the behavior change wheel to design a novel home‐based exercise program for adults living with overweight and obesity: Comprehensive reporting of intervention development
Source: Obes Sci Pract. 2024 Jun 19;10(3):e774. doi: 10.1002/osp4.774 (PMC11187404; doi:10.1002/osp4.774)
Supplement: Supplementary file 4 — Table S4 [file OSP4-10-e774-s001.docx]

Supplementary Material three

**Table 3**: Relationship between the COM-B, TDF and matching intervention functions most likely to be effective in bringing out that change.

| COM-B component | Theoretical Domains Frameworks | Intervention Functions |
| --- | --- | --- |
| Psychological capability | Knowledge | Education, Enablement |
|  | Cognitive and interpersonal skills |  |
|  | Memory |  |
|  | Attention and decision |  |
|  | Behavioural regulation |  |
| Physical capability | Physical skills | Education, Training, Modelling, Enablement |
| Physical opportunity | Environmental context and resources | Education, Training, Modelling  Enablement, Environment reconstructing |
|  |  |  |
| Social opportunity | Social influences | Training, Enablement |
| Reflective motivation | Social/professional role and identity | Education, Environment reconstructing |
|  | Beliefs about capabilities |  |
|  | Optimism |  |
|  | Intentions |  |
|  | Goals |  |
|  | Beliefs about consequences |  |
| Automatic motivation | Reinforcement | Enablement |
|  | Emotion |  |

COM-B: Capability, Opportunity, Motivation, Behaviour; TDF: Theoretical Domains Framework
